# Father’s perceptions and care involvement for their very preterm infants at French neonatal intensive care units

**DOI:** 10.3389/fpsyt.2023.1229141

**Published:** 2023-11-16

**Authors:** Amélie Stern-Delfils, Isabelle Leray, Laurence Caeymaex, Odile Dicky, Madeleine Akrich, Audrey Reynaud, Charlotte Bouvard, Anne Evrard, Jacques Sizun, Charlotte Tscherning, Pierre Kuhn, the GREEN Committee (Groupe de Réflexion et d’Evaluation de l’Environnement des Nouveau-nés de la Société Française de Néonatologie)

**Affiliations:** ^1^Department of Neonatology, Hospital of Mulhouse, Mulhouse, France; ^2^Department of Neonatology, University Hospital of Strasbourg, Strasbourg, France; ^3^NICU, Centre Hospitalier Intercommunal de Créteil, Créteil, France; ^4^Centre d’Etudes Discours Images Textes Ecrits Communication (CEDITEC), Paris Est Créteil University, Créteil, France; ^5^NICU, University Hospital, Toulouse, France; ^6^U1027 INSERM, Paul Sabatier University, Toulouse, France; ^7^Collectif inter-associatif autour de la naissance (CIANE), Paris, France; ^8^Association SOS Préma, Boulogne-Billancourt, France; ^9^NICU, Sidra Medicine Hospital, Well Cornell University Hospital, Doha, Qatar; ^10^Center for Pathophysiology Toulouse-Purpan (CPTP), Inserm Unviversity of Toulouse, Toulouse, France; ^11^Institut des Neurosciences Cellulaires et Intégratives, CNRS UPR, Strasbourg University, Strasbourg, France; ^12^Neonatal Research Unit, Department of Women’s and Children’s Health, Karolinska Institute, Stockholm, Sweden

**Keywords:** online survey administration, fathers, preterm infants, NICU, care involvement and presence

## Abstract

**Objectives:**

We aimed to evaluate (1) fathers’ perceptions and care involvement for their very premature infants and their views of the hospitalization period based on parental reports and (2) their evolution over time.

**Methods:**

We used an online parental survey to assess answers from parents of very preterm infants who were successfully discharged from French neonatal units. We analysed answers from February 2014 to January 2019 to an anonymous internet-based survey from the GREEN committee of the French Neonatal Society. Responses were compared for period 1 (P1, 1998 to 2013) and period 2 (P2, 2014 to 2019).

**Results:**

We analyzed 2,483 surveys, 124 (5%) from fathers and 2,359 (95%) from mothers. At birth, 1,845 (80%) fathers were present in the hospital, but only 879 (38%) were near the mother. The presence of fathers in the NICU increased from P1 to P2 (34.5% vs. 43.1%, *p* = 0.03). Nearly two thirds of fathers accompanied their infants during transfer to the NICU (1,204 fathers, 60.6%). Fathers and mothers had similar perceptions regarding relationships with caregivers and skin-to-skin contact with their infants. However, more fathers than mothers felt welcome in the NICU and in care involvement regarding requests for their wishes when they met their infant (79% vs. 60%, *p* = 0.02) and in the presentation of the NICU (91% vs. 76%; *p* = 0.03). Mothers and fathers significantly differed in the caring procedures they performed (*p* = 0.01), procedures they did not perform but wanted to perform (*p* < 0.001), and procedures they did not perform and did not want to perform (*p* < 0.01).

**Conclusion:**

Most fathers were present at the births of their very preterm infants, but fewer fathers were near the mother at this time. Less than two thirds of fathers accompanied their infants to the NICU. There should be further changes to better meet the specific needs of the fathers of infants requiring care in the NICU. Continuing assessment with an online questionnaire may be useful to monitor changes over time in father’s involvement in NICUs.

## 1 Introduction

The birth and hospitalization of a very preterm infant (VPI) in a neonatal intensive care unit (NICU) is a major disruption in the family’s life. The stressful and intimidating NICU environment and the uncertain health outcomes for newborns is especially traumatic for parents (1). The burden of these multiple stresses may have long-term consequences on the parent-child relationship and parents’ mental health (2). The parents of premature neonates have increased risk of depression, post-traumatic stress disorder, and anxiety (3–5). Infant-and family-centered developmental care strategies can prevent these complications and meet the family’s needs. Recent studies have recommended providing support for increased involvement of mothers and fathers in the care of their premature infants (6–8).

The presence of fathers in the NICU is now believed to promote the experience of fatherhood with the premature infant, increase the well-being of the mother and infant, and contribute to better infant brain development (8–10). Premature birth may reverse the roles of fathers and mothers in that fathers may be on the front line of care. In particular, fathers of such newborns often receive information on the infant’s health and communicate this information to their partners (11). However, often, fathers feel unable to care for their infants, lack self-confidence, and are intimidated by the small size and apparent fragility of their premature newborn (11–13).

Biological and neuroscientific studies have shown that fathers have an innate ability to bond with and care for their premature infants (14, 15). Several studies have shown that fathers of preterm infants wanted to play an active role in their infants’ care (9, 12, 16–19). Father-infant bonding appears to be facilitated by the development of fathering skills and increased involvement in infant care (9, 10).

Thus, fathers seeking to care for their premature newborns need support and guidance that meet their specific needs. Previous parental surveys have assessed parental experiences and needs in the NICU (20, 21). However, only a few studies have been performed at the national level, and they rarely specifically examined fathers’ views. A recent survey was submitted to parents in France (21, 22). This tool gave the opportunity to collect the presence and experiences of fathers’ in French NICUs.

Our main objective was to evaluate the perception of fathers’ presence at the time of birth of their VPIs and their experiences during their infants’ transfer to the NICU. Secondary objectives were to evaluate fathers’ experiences of feeling welcome in the NICU, their perceptions of their relationships with caregivers, and their participation in care and skin-to-skin contact, as compared to mothers’ experiences. Finally, we aimed to assess the evolution of these items over time.

## 2 Patients and methods

### 2.1 Development and distribution of the questionnaire

In France, an internet-based survey was started in February 2014 as a collaborative project between members of the French Neonatology Society and parental associations within the *Groupe de Réflexion et d*’*Evaluation du Nouveau-né* (GREEN Committee) (21, 22). The objective was to develop recommendations to improve family integration into NICUs and to modify the hospital environment so that it better meets the needs of parents and their infants (7).

This anonymous online survey for the parents of premature infants who were hospitalized in NICUs consists of 222 questions regarding neonatal care (some multiple-choice and some open-ended) and covers 9 distinct areas. The topics covered include the particular circumstances of the birth, the parent’s perceptions of feeling welcome, transfer of the infant to different units, breastfeeding, participation in care, and preparation for hospital discharge. It is intended for all parents who had a newborn infant hospitalized in a NICU.

### 2.2 Characteristics of parents and infants and data collection

**Figure 1:**
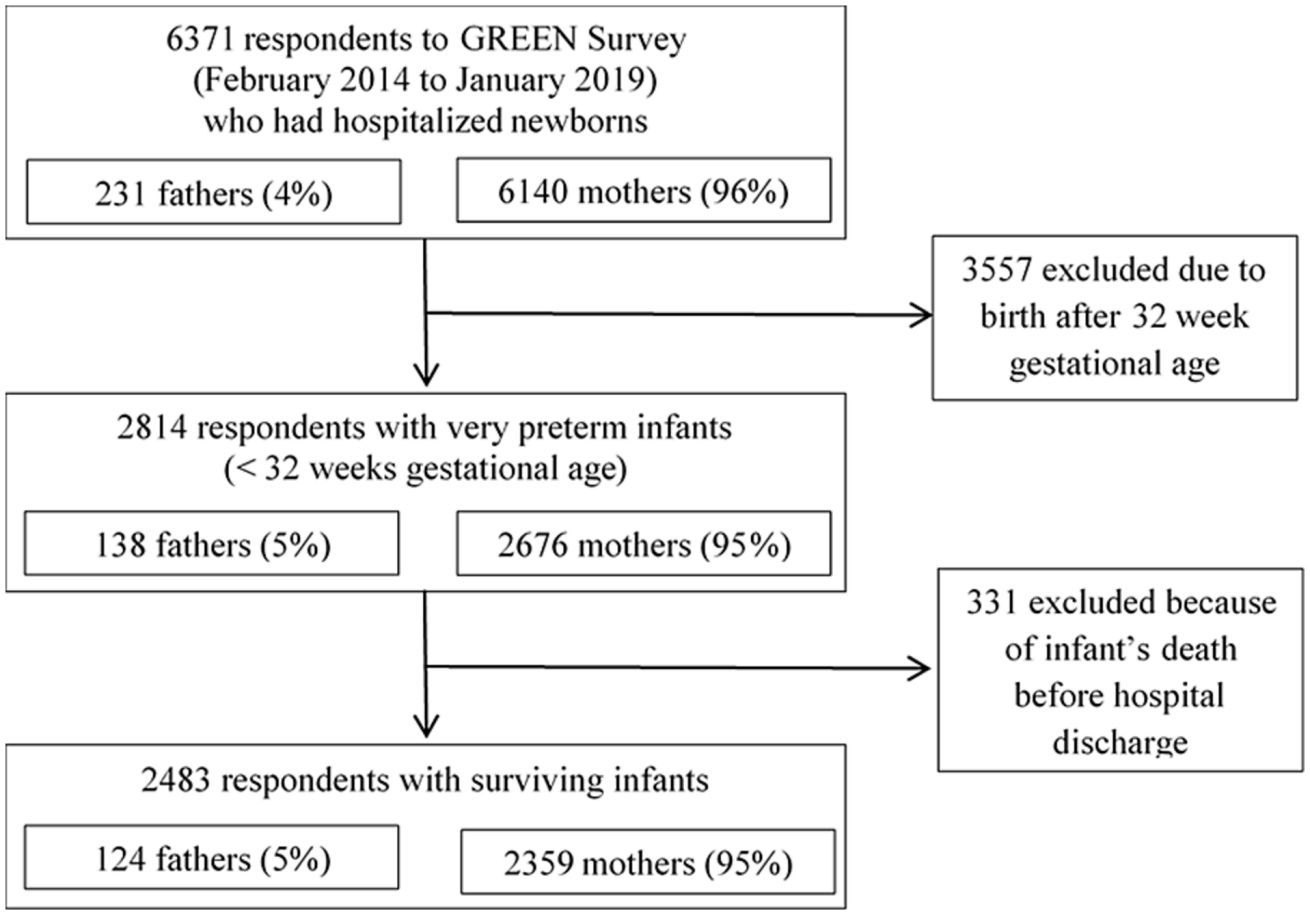
Study flow chart of the parents of very preterm infants who responded to the GREEN survey.

This study was an analysis of quantitative data based on responses collected up to January 2019. We focused on fathers whose infants were born before 32 weeks of gestational age and successfully discharged from the hospital (Figure 1).

A total of 29 items described the population of fathers as a whole: demographic characteristics, presence at the time of birth, and support provided during the infant transfer to the NICU. For these 29 items, we used the responses of all fathers and mothers.

A total of 34 items focused on both parents’ experiences. These allowed us to specifically analyse responses about feeling welcome in the NICU, relationships with caregivers, participation in different caring procedures (detailed in Table 1) and skin-to-skin contact. For these 34 questions, we compared the answers of mothers and fathers.

**Table 1:** Caring procedures.

Caring procedure domains	Type of caring procedures
Feeding	Preparing the milk in a syringe or cup Giving the bottle or cup Inserting or removing the nasogastric tube
Monitoring	Weighing/measuring your child Reporting monitoring data on your infant’s chart (oxygen saturation/heart rate) Taking your child’s temperature Turning off monitor alarms Putting electrodes on the chest
Hygiene care	Fully washing your child in the incubator Performing umbilical cord care Bathing your child wearing high flow or nasal CPAP cannula Bathing your child intubated
Diaper care and stools	Changing a nappy in the incubator Performing an abdominal massage Helping your child to stool by bending the knees
Positioning and clothing	Dressing your child wearing high flow or nasal CPAP cannula Dressing your child intubated Set up the cocoon or swaddling Cleaning the incubator or bed Cleaning feeding equipment (cup/bottle) Bringing clothes and sheets, washing them at home
Skin-to-skin contact or holding	Deciding when your unventilated child can be in skin-to-skin contact Deciding when your child can be in skin-to-skin contact with ventilation or nCPAP Deciding when your unventilated child can be held Deciding when your child can be held with ventilation or nCPAP Deciding when your intubated child can be held
Medical care	Putting on the cannula and repositioning the child in phototherapy Giving glucose before a painful procedure Hold the child by swaddling and comforting during a painful procedure Stimulating the child in case of apnoea Changing O2 high flow cannula Changing the CPAP equipment Suctioning at the nose or mouth
Administering pharmacological treatment	Administering medications orally Administering medications through the gastric tube Putting in eyedrops Administering inhalation

CPAP, continuous positive airway pressure; nCPAP, nasal continuous positive airway pressure.

Finally, we compared the periods of VPI birth from 1998 to 2013 (period 1; P1) and 2014 to 2019 (period 2; P2) to analyse changes over time as the first recommendations of the GREEN committee, aiming to improve family integration into NICUs, were presented in 2014.

Moreover, open-ended questions were included in the survey to allow the expression of individual feelings of the responders. They were used to describe more precisely the father’s perception and to illustrate the presentation of the results of the survey with individual testimonies about fathers’ care involvement.

### 2.3 Statistical analysis

Quantitative variables are presented as mean and standard deviation (SD) or number (%) and were compared by student *t*-test or Fisher’s exact test, as indicated. For multiple comparisons, the *p*-value was adjusted using Holm’s method (23). To compare the proportions of fathers and mothers among numerous items, we used the signs test (24). All statistical analyses were performed with R v3.5.2.

## 3 Results

Table 2 shows the characteristics of responding parents and their VPIs. Significantly more fathers than mothers reported living as couples, fathers had significantly more education than mothers, and the infants of fathers were significantly more preterm. As compared with P1 (1998 to 2013), during P2 (2014 to 2019), fathers were older [mean age 32.7 (5.6) vs. 31.3 (5.9) years, *p* < 0.0001], more families were living as couples (94.2% vs. 89.4%; *p* < 0.0001), and the proportion of families with siblings was greater (35.3% vs. 29.8%, *p* < 0.01) (data not shown). Not all fathers and mothers responded to all the questions of the survey. The number of responders is specified for each question when it is necessary.

**Table 2:** Characteristics of respondents and their infants.

Characteristics	Fathers’ responses (*N* = 124)	Mothers’ responses (*N* = 2,359)	*p*
Father’s age, mean (SD), *N* = 2,414	32.6 (5.6)	31.9 (5.8)	0.19
Mother’s age, mean (SD), *N* = 2,478	31.6 (5.1)	30.8 (5.4)	0.1
Mother’s family situation at the time of birth: in couple, *n* (%), *N* = 2,483	124 (100)	2,256 (95.6)	**<0.01**
Mother’s current family situation: in couple, *n* (%), *N* = 2,483	118 (95.2)	2,155 (91.4)	0.18
Father’s education level, *n* (%), *N* = 2,402			**<0.001**
College level	37 (29.8)	1,084 (47.6)	
Baccalaureate level	1 (0.8)	103 (4.5)	
Higher education	86 (69.4)	1,091 (47.9)	
Mother’s education level, *n* (%), *N* = 2,482			**<0.05**
College level	30 (24.2)	810 (34.4)	
Baccalaureate level	1 (0.8)	59 (2.5)	
Higher education	93 (75)	1,489 (63.1)	
Father’s occupation, *n* (%), *N* = 2,409			0.63
Employed	121 (97.6)	2,180 (95.4)	
Unemployed	3 (2.4)	98 (4.3)	
Student	0 (0)	4 (0.2)	
Other (pensioner etc.)	0 (0)	3 (0.1)	
Mother’s occupation, *n* (%), *N* = 2,482			0.17
Employed	109 (87.9)	1,961 (83.2)	
Unemployed	13 (10.5)	380 (16.1)	
Student	2 (1.6)	15 (0.6)	
Other (pensioner etc.)	0 (0)	2 (0.1)	
Father’s native language, *n* (%), *N* = 2,418			0.1
French	118 (95.9)	2,077 (90.5)	
Other language	1 (0.8)	83 (3.6)	
Bilingual, including French	4 (3.3)	135 (5.9)	
Mother’s native language, *n* (%), *N* = 2,483			0.08
French	114 (92)	2,220 (94.1)	
Other language	5 (4)	32 (1.4)	
Bilingual, including French	5 (4)	107 (4.5)	
Single or multiple births, *n* (%), *N* = 2,483			0.27
Singleton	103 (83.1)	1,988 (84.3)	
Twins	20 (16.1)	351 (14.9)	
Triplets	0 (0)	17 (0.7)	
Quadruplets	1 (0.8)	3 (0.1)	<0.05
Infant’s gestational age at birth, median (min–max), *N* = 2,483	28 (24-31)	29 (24-31)	**<0.05**
Birth weight, g, *n* (%), *N* = 2,483			0.37
<500	1 (0.8)	33 (1.4)	
500 to 1,500	113 (91.1)	2,002 (84.9)	
>1,500	10 (8.1)	324 (13.7)	
Family with siblings living at time of birth, *n* (%), *N* = 2,480	43 (34.7)	757 (32.1)	0.56
Number of hospitals where the infant received care, median (min–max), *N* = 2,282	1 (1–5)	1 (1–5)	0.36

Bold values indicate statistically significant differences.

### 3.1 Presence of fathers at birth and during transfer to the NICU

Most fathers were present at birth, but less than half were near the mother, mainly due to medical restrictions.

The fathers regretted not being at their wives’ side: “*They did not come to get me to attend the cesarean*” or “*The nurses initially took my wife. After 3 min that seemed like an eternity*, *I had to insist twice and impose myself to attend the delivery. Our children arrived a few minutes later*.”

However, the father’s presence near the mother increased over time. Most fathers were separated from their infants less than 24 h. Fathers visited before mothers. An increasing majority of fathers accompanied their infants to the NICU and most fathers accompanied their infants during intra-hospital transfers with caregivers.

The transfer of the newborn to the NICU remained a moment of particular vulnerability: the lack of information, the lack of welcome time and the feeling of loneliness were difficult for fathers: “*My twins were taken to the unit without me. When I arrived*, *the doctors and nurses were all busy caring for my twins. So no one greeted me*” or “*When he was born*, *we could not see him; he was immediately taken to the next room to be intubated. We did not hear him scream*” or “*I wish I had accompanied my child to NICU. I found myself without my baby and without my wife*, *alone and distraught. It was a very difficult time for me*.”

The fathers reported easily finding the location of the NICU, even when going alone. This was similar for inter-hospital transfer, which fathers mostly performed on their own. In comparing P1 to P2 (Table 3), the father’s presence near the mother at birth increased over time.

**Table 3:** Comparison of fathers’ presence at birth and transfer of newborns to other hospital units during period 1 (P1; 1998–2013) and period 2 (P2; 2014–2019).

	Total (*N* = 2,483)	Overall population from P1 (1998–2013) (*N* = 1,371)	Overall population from P2 (2014–2019) (*N* = 1,112)	Adjusted *p*
Father at birth was, *n* (%), *N* = 2,291				0.03
Present in birth room with mother	879 (38.4)	439 (34.5)	440 (43.1)	
Present but in a room next door because not allowed in birth room by medical staff	932 (40.7)	543 (42.7)	389 (38.1)	
Present but in a room next door, because did not want to attend	34 (1.5)	21 (1.7)	13 (1.3)	
Not present because of no time or unable to come	318 (13.9)	196 (15.4)	122 (12)	
Not present for other reasons	128 (5.5)	72 (5.7)	56 (5.5)	
Length of time between birth and mother’s first visit with baby, *n* (%), *N* = 1,960				0.001
No separation	130 (6.6)	58 (5.3)	72 (8.4)	
Separation less than 24 h	1,330 (67.9)	701 (63.7)	629 (73.1)	
Separation more than 24 h	500 (25.5)	341 (31)	159 (18.5)	
Length of time between birth and father’s first visit with baby, *n* (%), *N* = 2,009				0.15
No separation	795 (39.6)	420 (37)	375 (42.8)	
Separation less than 24 h	1,148 (57.1)	667 (58.8)	481 (55)	
Separation more than 24 h	66 (3.3)	47 (4.2)	19 (2.2)	
Number of fathers who accompanied baby to the first neonatal unit, *n* (%), *N* = 1,986	1,204 (60.6)	648 (57.9)	556 (64.2)	0.19
Means by which father followed his baby to another hospital, *n* (%), *N* = 73				1
By himself	71 (97.3)	46 (97.9)	25 (96.2)	
In an ambulance	2 (2.7)	1 (2.1)	1 (3.8)	
If father went by himself, those who easily found the service where the infant was transferred, *n* (%) (*N* = 58)	53 (91.4)	32 (91.4)	21 (91.3)	1
For infant’s transfer in the same hospital, fathers who were accompanied by a member of the healthcare team, *n* (%), *N* = 1,042	901 (86.5)	455 (83.5)	446 (89.7)	0.17
If fathers went there alone, those who easily found the service where the infant was transferred, *n* (%), *N* = 132	115 (87.1)	74 (89.2)	41 (83.7)	1

### 3.2 Feeling welcome in the NICU

Overall, parents felt very welcome by the medical team, they reported that they received adequate and easily understood information, and the efficiency of the healthcare workers made them trust the team (see Tables 4, 5). More fathers than mothers had positive feelings during the first visit and during explanations of the unit’s guidelines. Mothers met physicians later than did fathers but not significantly.

**Table 4:** Parents’ perceptions of feeling welcome in neonatal units.

	Fathers	Mothers	Adjusted *p*
Upon arrival in the neonatal care intensive unit, *n* (%)			
The parent felt expected by the medical team (*N* fathers = 95 and *N* mothers = 1,944)	83 (87)	1,606 (83)	1
The team devoted the time required (*N* fathers = 95 and *N* mothers = 1,944)	93 (98)	1,731 (89)	0.49
The team asked about the parents’ wishes/needs when meeting with the infant (*N* fathers = 86 and *N* mothers = 1,880)	68 (79)	1,128 (60)	0.02
During this first visit, the parent was introduced to, *n* (%) (*N* fathers = 95 and *N* mothers = 1,944)			
The service and its operating rules	87 (92)	1,474 (76)	0.03
The caregivers of the infant	85 (89)	1,597 (82)	1
The infant’s room and with explanations about the function of each device	81 (85)	1,497 (77)	1
The infant	85 (89)	1,720 (88)	1
Waiting time before talking with a member of the healthcare team (nurse, caregiver, intern, physician) about the condition of infant, *n* (%) (*N* fathers = 100 and *N* mothers = 1,960)			
After 24 h	6 (6)	294 (15)	0.56
Waiting time before meeting a physician, *n* (%) (*N* fathers = 91 and *N* mothers = 1,731)			
After 24 h	20 (22)	658 (38)	0.08
During the first interview with the physician, the parent felt, *n* (%) (*N* fathers = 95 and *N* mothers = 1,890)			
Supported	83 (87)	1,561 (83)	1
Listened to	86 (91)	1,647 (87)	1
Included in the practice	82 (86)	1,536 (81)	1
The explanations given at that time were, *n* (%) (*N* fathers = 95 and *N* mothers = 1,930)			
Understandable	83 (87)	1,702 (88)	1
Adequate	77 (81)	1,424 (74)	1
These explanations were, *n* (%)			
Too technical (*N* fathers = 92 and *N* mothers = 1,870)	12 (13)	227 (12)	1
Of an appropriate technical level (*N* fathers = 92 and *N* mothers = 1,870)	76 (83)	1,533 (82)	
Insufficiently technical (*N* fathers = 100 and *N* mothers = 1,975)	5 (5)	79 (4)	
This interview generally gave confidence to the team, *n* (%) (*N* fathers = 95 and *N* mothers = 1,830)	87 (92)	1,683 (92)	1

**Table 5:** Fathers’ words about their perception and their welcome in the NICU.

Negative feelings	Surprised and lost	“*In the hours following the birth*, *one is* ‘*stunned*’.” “*The transfer from the NICU was abrupt and this memory will remain one of the most striking*”
	Not needed “technical” explanations	“*No impatience about the technical questions*, *which I knew I would have time to ask later*, *and if possible with the mother*”
	Ambivalence between health to the newborn and mother	“*Big ambivalence*, *between the fact of needing to accompany and be reassured on the state of the baby*, *and the anxiety of leaving the mother alone in the birth room*”
	Feeling of loneliness and anxiety in the absence of welcome	“*And when we arrived in the NICU*, *not a glance*, *not a word*, *we were spectators of the placement of our baby in incubator*, *that seemed so abrupt that we cried. For many minutes*, *everyone was around the incubator*, *without anyone noticing our presence*”
Positive supports	A human and benevolent welcome	“*The team was waiting for us*” “*The reception in the unit was of an exceptional quality. At the same time*, *it was dedramatizing*, *human*, *empowering and involving*”
	Parental involvement with feeling as “care partner”	“*I was taken into account*” “*They asked me what I wanted with my wife*” “*They said that we had a great role to play*” “*They involved us in every decision*, *and as a father*, *it feels good to be able to take on that role*, *they gave me room.*” “*The doctor immediately named me as Robin*’*s father*, *thus helping me to take this place for my son. This was beneficial and involving.*”
	A team that takes the time and answers questions, in a comprehensible medical language	“*They never tried to hide information from us*, *they took our concerns into account*, *our questions were always answered.*” “*The nurse and the pediatricia*, *took the time to talk to us*, *to answer our questions and to talk about what was next without lying to us and without falling into incomprehensible medical language.*”

### 3.3 Relationships with caregivers

Overall, parents were very satisfied or satisfied about their relationships with caregivers. Most fathers and mothers perceived the staff as being available and felt confident in asking questions. They also felt involved in decision-making about the infant’s well-being, health, and daily schedule. The parents felt comfortable talking openly to the caregivers, and the information they received seemed consistent among different caregivers. Fathers and mothers did not significantly differ in caregiver relationships (see Tables 6, 7).

**Table 6:** Relationships of parents and caregivers.

	Fathers	Mothers	Adjusted *p*
Relationships with caregivers were satisfactory or very satisfactory, *n* (%)			
In neonatal intensive care unit (*N* fathers = 90 and *N* mothers = 1,795)	88 (98)	1,768 (98)	1
In neonatal ward (*N* fathers = 85 and *N* mothers = 1,795)	83 (98)	1,585 (88)	1
In kangaroo unit (*N* fathers = 23 and *N* mothers = 500)	23 (100)	440 (88)	1
Relationships with the health care team, *n* (%) (*N* fathers = 82 and *N* mothers = 1,736)			
Staff was available when parents requested them	74 (90)	1,495 (86)	1
Parents felt confident with the staff who cared for the infant	73 (89)	1,509 (87)	1
Parents felt comfortable asking questions or for clarification when explanations were unclear	67 (82)	1,425 (82)	1
Overall, explanations and parents’ involvement were possible in decision-making regarding, *n* (%) (*N* fathers = 82 and *N* mothers = 1,728)			
The infant’s well-being	68 (83)	1,452 (84)	1
The infant’s health (treatment, ventilation, nutrition)	69 (84)	1,387 (80)	1
The organization of the infant’s day (care schedules, skin-to-skin contact, etc.)	71 (87)	1,407 (81)	1
The team considered the parent’s comments about the infant’s health and well-being, *n* (%) (*N* fathers = 78 and *N* mothers = 1,690)	63 (81)	1,301 (77)	1
Overall, the information was consistent from one person to another, *n* (%) (*N* fathers = 82 and *N* mothers = 1,715)	67 (82)	1,372 (80)	1
The parent felt comfortable speaking openly to the healthcare team, *n* (%) (*N* fathers = 82 and *N* mothers = 1,700)	64 (78)	1,224 (72)	1
The reasons why they did not feel comfortable talking with the health care team, *n* (%)			
Difficulty in formulating their requests (*N* fathers = 5 and *N* mothers = 131)	4 (80)	105 (80)	1
Shyness (*N* fathers = 6 and *N* mothers = 159)	5 (83)	135 (85)	
Fear of being judged (*N* fathers = 6 and *N* mothers = 166)	5 (83)	141 (85)	
Lack of availability of the healthcare team (*N* fathers = 6 and *N* mothers = 173)	5 (83)	152 (88)	
Fear that openness will influence caregivers’ relationships with the infant (*N* fathers = 7 and *N* mothers = 219)	7 (100)	197 (90)	
Possibility of attending physicians’ visits to the infant, *n* (%) (*N* fathers = 83 and *N* mothers = 1,725)	62 (75)	1,190 (69)	1
It helped the parent (*N* fathers = 59 and *N* mothers = 1,142)	50 (85)	959 (84)	1
The parent missed it (*N* fathers = 21 and *N* mother s = 496)	17 (81)	372 (75)	1

**Table 7:** Fathers’ feelings and words expressed about caregivers.

Positive feelings “What helped you the most during the newborn’s hospitalization?”	“*Humanity*” “*Kindness*” “*Smile*” “*Professionalism*” “*Benevolence*” “*Moral support and comfort*” “*Availability*” “*Know-how*” “*Listening*” “*Involvement*” “*Reassurance,*” “*Presence and support*” “*Gentleness*” “*Empathy*”
Negative feelings	“*We were not really parents*” “*Under the control and judgment of the caregivers*” “*Caregivers contradicted each other in their methods*” “*We are nothing*, *not even the parents*, *nothing is chosen or asked of us*” “*Some caregivers purposely keep parents out of their child*’*s care by telling us that we do not know how to do it*” “*No communication about the risks*, *the tests performed on the baby. We feel like spectators or even on the sidelines*, *powerless and passive*” “*Preference given to the mother systematically. Father*’*s opinion not often asked*”

### 3.4 Participation in care

Overall, most parents felt very positive regarding the support they received from caregivers for participating in their infants’ care (Table 8). We also analysed participation in care more precisely by identifying different caring procedures (see Table 1).

**Table 8:** Participation of parents in care of their infants.

	Fathers	Mothers	Adjusted *p*
Concerning the infant’s care, *n* (%) (*N* fathers = 78 and *N* mothers = 1,580)			
Staff offered to participate in care as soon as possible	73 (94)	1,505 (95)	1
The first time parents were involved in care, the staff gave them confidence and accompanied them	76 (97)	1,493 (95)	1
Opportunity to take on the role of parent	67 (86)	1,285 (81)	1
Feeling of being judged by, *n* (%)			
Nurses (*N* fathers = 80 and *N* mothers = 1,606)	8 (10)	273 (17)	1
Physicians (*N* fathers = 83 and *N* mothers = 1,555)	5 (6)	171 (11)	1
Caregivers were able to respect preferences for the care that parents wanted to provide, *n* (%) (*N* fathers = 75 and *N* mothers = 1,530)	66 (88)	1,316 (86)	1
			
Caregivers were able to respect the time parents needed before they began to participate in their infant’s care, *n* (%) (*N* fathers = 78 and *N* mothers = 1,572)	70 (90)	1,368 (87)	1

**Figure 2:**
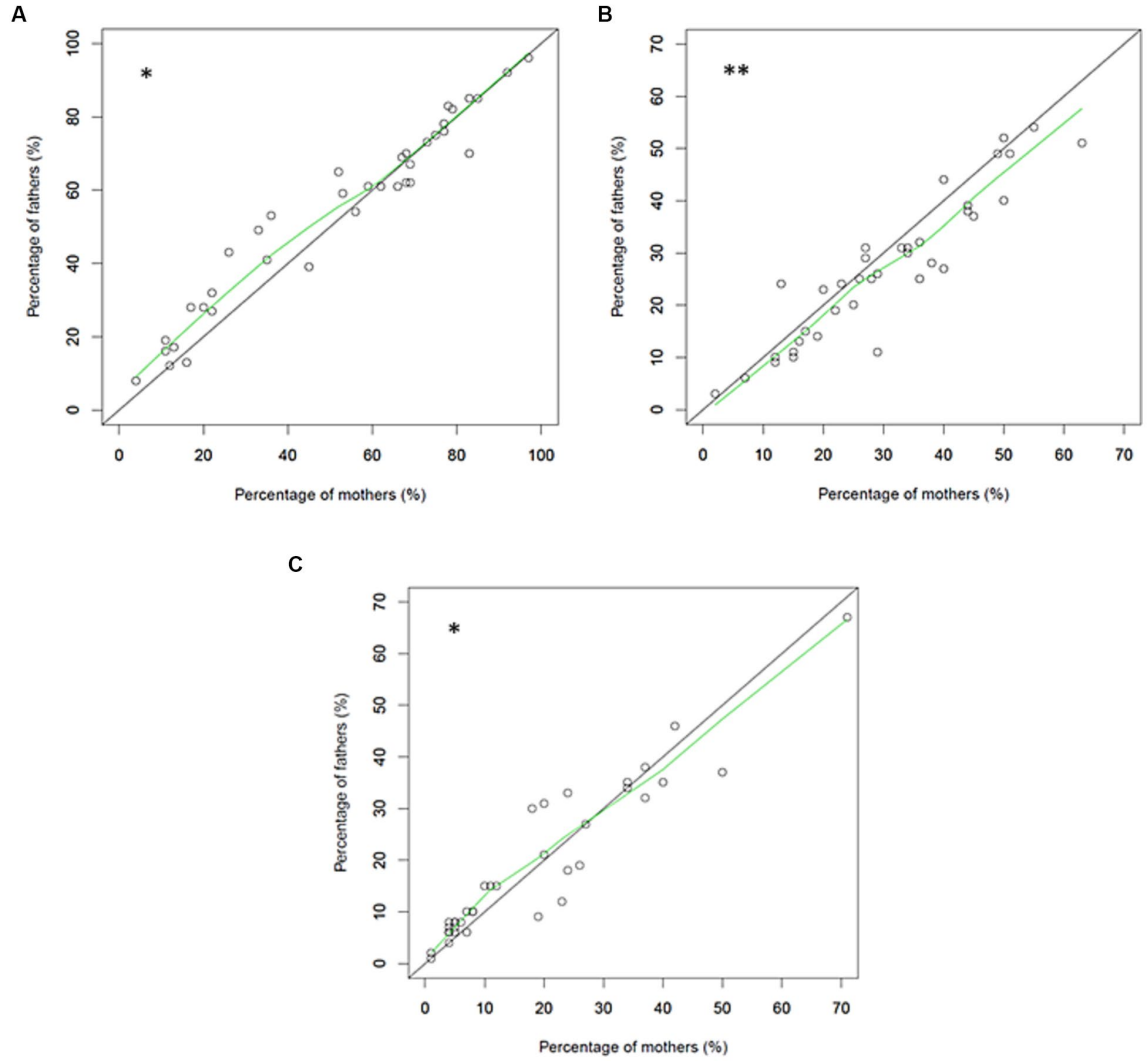
Relationships of the percentages of mothers and fathers who performed different caring procedures **(A)**, who did not perform different caring procedures that they wanted to perform **(B)**, and who did not perform different caring procedures that they did not want to perform **(C)**.

However, when comparing caring procedures overall between mothers and fathers, we found significant differences in the number of caring procedures that were performed, procedures not performed but that the parent wanted to perform, and procedures not performed and the parent did not want to perform (Figure 2). Overall, fathers performed more caring procedures than mothers (Figure 2A; *p* = 0.01), including bathing and dressing the intubated infant, preparing the infant for phototherapy, and stopping the monitor alarms. More mothers than fathers wanted to perform caring procedures that they did not actually perform (Figure 2B; *p* < 0.001), including cord care, bathing and dressing of the intubated infant, preparing for breast feeding, and administering eye drops. Finally, more fathers than mothers reported not performing caring procedures that they did not want to perform (Figure 2C; *p* < 0.01), including washing the infant completely in an incubator, administering a sweet solution, and performing facilitated tucking during a painful procedure. However, more mothers than fathers did not perform certain other caring procedures that they did not want to perform, including installing and removing a nasogastric tube and performing nasal or oral suction procedures.

Fathers and mothers did not differ in specific caring procedures, except for care of the umbilical cord, with significantly more mothers than fathers not performing cord care although they wanted to (29% vs. 11%, *p* = 0.026).

### 3.5 Participation in skin-to-skin contact

Most parents were informed of the benefits of skin-to-skin contact, performed this procedure, and felt confident when doing so: “*Skin-to-skin contact was very strongly recommended to us*, *which we obviously agreed with* (see Table 9). *We had a comfortable chair*, *available staff*, *everything was always done with patience*, *with measured and careful gestures*.” Fathers and mothers did not significantly differ in this care, especially regarding the timing of the first skin-to-skin contact. However, a higher proportion of mothers than fathers would have liked to perform skin-to-skin contact more often.

**Table 9:** Participation of parents in skin-to-skin contact with their infants.

	Fathers	Mothers	Adjusted *p*
Number of parents informed about the benefits of skin-to-skin contact, *n* (%) (*N* fathers = 74 and *N* mothers = 1,530)	70 (95)	1,443 (94)	1
			
Either parent was able to practice skin-to-skin contact with the infant at least once, *n* (%) (*N* fathers = 74 and *N* mothers = 1,530)			
No, neither parent did	1 (1.3)	26 (1.7)	0.002
Yes, the other parent did	5 (6.8)	12 (0.8)	
Yes, both parents did	63 (85)	1,242 (81)	
Yes, I did	5 (6.8)	267 (17)	
Infant’s age when they first had skin-to-skin contact, *n* (%) (*N* fathers = 68 and *N* mothers = 1,510)			
1 to 3 days	28 (41)	652 (43)	1
3 to 7 days	23 (34)	441 (29)	
1 to 2 weeks	4 (6)	195 (13)	
2 to 3 weeks	7 (10)	100 (7)	
> 3 weeks	6 (9)	121 (8)	
Concerning this first skin-to-skin contact, the parent thought that, *n* (%) (*N* fathers = 68 and *N* mothers = 1,468)			
It was the right time	59 (87)	1,128 (77)	0.63
It was offered too soon	1 (1.5)	51 (3.5)	
It was offered too late	7 (10)	296 (20)	
Number of parents who would have liked to have skin-to-skin more often, *n* (%) (*N* fathers = 65 and *N* mothers = 1,468)	37 (57)	1,087 (74)	0.026
			
The duration of skin-to-skin contact times was generally determined by, *n* (%) (*N* fathers = 68 and *N* mothers = 1,510)			
Parents	17 (25)	358 (24)	1
Nurse	19 (28)	329 (22)	
Physician	3 (4.4)	165 (11)	
Mutual agreement between the health care team and parents	5 (7.4)	83 (5.5)	
The infant’s state of health	24 (35)	572 (38)	
Number of parents who felt confident during skin-to-skin contact, *n* (%) (*N* fathers = 68 and *N* mothers = 1,505)	65 (95.6)	1,404 (93)	1
			
Parents’ preference to have their infant, *n* (%) (*N* fathers = 68 and *N* mothers = 1,508)			
In skin-to-skin contact	6 (9)	80 (5.3)	1
In their arms	48 (71)	1,160 (77)	
No preference	14 (21)	267 (18)	

Nonetheless, some fathers’ open-ended comments about skin-to-skin contact described a stressful experience: “*For the first skin-to-skin contact*, *the nurse insisted when I wasn’t ready; it was too early for me*, *I was tired and was afraid he would breathe wrong on me*” or “*During skin-to-skin contact*, *the temperature was dropping too fast*, *and I was afraid I was making him worse*.” or “*I felt more uncomfortable than insecure*, *a little ridiculous unclothed in the nursing unit*” or “*I was afraid of respiratory arrest when the assistants were removed and afraid of falling asleep with the baby and not doing it right*.”

## 4 Discussion

The results of this national web-based survey indicated that most fathers were present at birth, but less than half were near the mother at this time, although this number increased slightly over time. Very few fathers reported no separation from their infants, although most met their VPIs within 1 day of birth. Less than two thirds of fathers accompanied their infants during transfer to the NICU. We found many similarities between the responses of fathers and mothers; however, there were significant differences in the perceptions of fathers and mothers regarding feeling welcome in the neonatal unit and involvement in care of their infants.

The main limitation of this study was that it used an internet-based open-access questionnaire that required knowledge of the French language, which probably explains why the study population had a high percentage of parents with high socio-economic class and traditional family structure. Thus, our results may not be applicable to fathers from economically vulnerable families. In addition, our study population probably had more fathers that were involved in their infant’s care. In agreement, a European study also reported that fathers with higher education were more likely to be present in the unit during medical rounds than other fathers (25). This situation could limit the generalizability of our results regarding fathers’ perceptions. In addition, because our data were collected retrospectively, there was a risk of incorrect reporting of information by parents, whose perceptions could have changed over time and after discharge. Data were also missing for some items in the questionnaire for some respondents. However, the very high number of total answers from mothers regarding the fathers’ behaviors suggests the validity of our data.

To our knowledge, this was the first nation-wide study that used quantitative data to assess fathers’ presence at the birth of their preterm infants and during their transfer to the NICU. Previous studies have reported fathers’ perceptions during pregnancy and birth (26) but not the proportions of fathers present at birth and the presence of the father with the mother at that time. Similar to our results, a German survey from 2011 interviewed 111 fathers of very low birth weight infants in 2 NICUs and found that nearly all the fathers met their infants on the first day of life. However, first contact was earlier for these German fathers than the fathers in our study in that 33.3% of them saw their infants at birth and 61.3% saw them within 1 h of birth (27).

There is a general lack of data regarding the presence and role of fathers at birth of a VPI and during transfer to the NICU, even though fathers reported being very satisfied when accompanying their infants immediately after birth and when encouraged to touch and hold them (18). Current recommendations for infant-and family-centered developmental care strongly support parents being as close as possible to their infants (6) and that separation should be avoided. European studies showed that policies regarding parental presence and involvement in the NICU varied widely among countries and among NICUs within individual countries (28, 29). There is greater support for parental presence in the NICU in northern than other European countries. However, increasing emphases are being placed on applying fewer restrictions for parents regarding access to the NICU, more encouragement for parents to provide skin-to-skin contact, and improved accessibility of parents to bedrooms, family kitchens, and private bathrooms in NICUs (28, 30). These changes of NICU policies are essential for successfully promoting a father’s physical proximity with their infant. Father-infant closeness appears to be important in fostering the father-infant bond (8), mainly through care involvement and skin-to-skin contact (9, 11, 12, 31–33).

We found slight differences between fathers and mothers regarding their perceptions of feeling welcome in the NICUs and involvement in their infants’ care. No previous studies have provided accurate comparisons of self-reported fathers’ and mothers’ involvement in the care of preterm infants. A few studies compared maternal and paternal perceptions on this topic, but these were not nation-wide studies and very few parents were interviewed (18, 31, 32, 34, 35). Some other studies on the topic considered only the father’s views (9, 13, 16, 17, 19, 27, 33, 36). Altogether, these studies indicated that fathers needed explanations about the function of the NICU and its equipment and about the baby’s sensory and relational abilities (31, 34, 36). Fathers reported feeling the need to be included early in care and to feel valued as a parent (35), in accordance with our results.

The responding fathers in our study were also very motivated to participate in care, notably expressing a desire to perform caring procedures that typically only a minority of parents perform. Moreover, fathers performed more technical procedures than mothers in our study. Interpreting this difference was difficult because (1) our study design did not allow for comparing mothers and fathers of the same infant; (2) we recruited parents from a large number of NICUs, which may differ in their parental integration policies; and (3) we could not exclude selection bias that favored the participation of fathers who wanted to participate in care (32). However the architectural design of a NICU and the level of implementation of infant-and family-centered care strategies could increase the involvement of parents and the frequency of skin-to-skin contact, particularly among fathers (25). In this case, the healthcare team plays an essential and supportive role (11, 16, 31, 35) in building trust and putting fathers at ease when providing care, such as skin-to-skin contact, and helping them be more involved with infant care (27, 33).

The need for fathers to be present near their partners and to be involved in infant care requires changes such as provision of supportive policies in the NICU and encouragement from healthcare teams of both parents to participate in their infants’ care (31). Caregivers can have a decisive influence on fathers (31) and should be aware of the barriers that fathers have described such as the newborn’s physical appearance, the technical NICU environment, and changes in parental roles, which may lead them to feel they cannot adequately care for their infant (11–13). Caregivers must get to know each parent, learn about the type of involvement they want (13), and estimate the time they need before providing care (9, 32). The Family Initiative’s International Neonatal Fathers Working Group drafted 12 practical recommendations to be used by neonatal teams to support the development of father-infant bonds and enable fathers to experience more equal co-parenting. These recommendations included assessing the needs and wishes of the father; ensuring flexibility and ease of access to the neonatal unit for fathers; providing information about infants directly to fathers, not exclusively through mothers (37); and providing information in real time (16, 35). Research has recommended encouraging the presence and care involvement of fathers and supporting them during their transition to fatherhood (31, 37). Other research reported that fathers felt the need to meet with other fathers who would better understand their own fears and difficulties (37). This could be facilitated by the establishment of discussion groups for fathers. Some authors have proposed the creation of interactive social media support that provides fathers with electronic updates on their infants’ health condition and allows communication with other fathers (35).

One highly effective procedure is to encourage the presence of fathers in family rooms during the entire hospitalization period, a strategy with proven short-term benefits for the infant (38). This approach is also supported by the implementation of infant-and family-centered care, which has widespread social support at the national level (39). European countries have disparities in the social support and parental leave policies provided to new parents (40), with Nordic countries providing the most generous benefits. However, most member states of the European Union now provide statutory parental leave (41). They aim at providing greater support for fatherhood an promoting more gender equality. The European Standards of Care for Newborn Health called for continuous parental support and access as well as high parental involvement in the care of newborns (42). There are similar recommendations in France (7), and the laws in France regarding social support for fathers of very preterm infants have evolved. Since July 1, 2019, all fathers whose newborn infants require immediate hospitalization at birth receive 30 days of paternity leave (43) in addition to the 25 days allocated to all fathers since July 2021 (44). Before that last date, and at the time of the survey, only 11 days were allocated. Fathers in France are now able to spend more time with their preterm infants. In view of the demonstrated benefits of father’s involvement in the care of their very preterm infants through early interventions, there is a need to further support this evolution in every country (45).

Large prospective studies are necessary to evaluate fathers’ presence and perceptions regarding their very preterm newborns. Quantitative studies should measure actual involvement during the whole duration of hospitalization, and qualitative studies should assess the feelings and needs of fathers. Future research should also examine the mothers and fathers of VPIs who have lower socioeconomic status because these parents may have different needs.

## 5 Conclusion

Most fathers were present at the births of their VPIs, but less than half were near the mother at this time. Only a small number of fathers reported no separation from their infants, although most met their infants during the first day of life. Less than two thirds of the fathers accompanied their infants on transfer to the NICU. Altogether, these data indicate room for improvement to meet the specific needs of fathers in the broad context of infant-and family-centered developmental care for premature infants. The continuation of this online questionnaire will allow for assessing future progress.

## Data availability statement

The raw data supporting the conclusions of this article will be made available by the authors, without undue reservation.

## Ethics statement

The studies involving humans were approved by Comité d’Ethique de la Faculté de Médecine et d’Odontologie de l’Université de Strasbourg. The studies were conducted in accordance with the local legislation and institutional requirements. Written informed consent for participation was not required from the participants or the participants’ legal guardians/next of kin in accordance with the national legislation and institutional requirements.

## Author contributions

AS-D analyzed the data, reviewed the literature, and wrote the first draft of the manuscript. IL conducted the extraction and the analyses of the data and generated the figures and tables of the manuscript. LC, OD, and JS discussed the interpretation of the results and reviewed the manuscript. MA, AR, CB, and AE contributed actively to the conceptualisation, the accessibility and dissemination of the GREEN parental questionnaire. CT supervised the conceptualisation and dissemination of the GREEN parental questionnaire and discussed the results, and reviewed the manuscript. PK contributed to the conceptualisation of the GREEN questionnaire, designed the study, supervised the analyses, and thoroughly reviewed the final manuscript. All authors contributed to the article and approved the submitted version.

## Group members of the GREEN Committee (Groupe de Réflexion et d’Evaluation de l’Environnement des Nouveau-nés de la Société Française de Néonatologie)

Aurore Allen (Port Royal-Paris), Frédérique Berne-Audeoud (CHU Grenoble), Charlotte Bouvard (SOS Préma), Anne Brandicourt (CH Sud Francilien), Charlotte Casper (CHU Toulouse), Laurence Caeymaex (CHIC Créteil), Hélène Denoual (CH Le Mans), Marie Agnès Duboz (CHU Besançon), Anne Evrard (Comité Inter-Associatif de la Naissance), Christine Fichtner (CHU Saint-Etienne), Céline Fischer-Fumeaux (CHUV Lausanne) Laurence Girard (Association Connaître), Françoise Gonnaud (CHU Lyon), Dominique Haumont (Hôpital Saint-Pierre Bruxelles), Petra Hüppi (CHU Genève), Nadine Knezovic (CHU Strasbourg), Pierre Kuhn (CHU Strasbourg), Elisabeth Laprugne-Garcia (CHU Lyon), Sophie Legouais (Paris), Fabienne Mons (CHU Limoges), Valérie Pelofy (CHU Toulouse), Jean-Charles Picaud (CHU Lyon), Véronique Pierrat (CHU Lille, Inserm EPopé), Patrick Pladys (CHU Rennes), Audrey Renaud (SOS préma), Laurent Renesme (CHU Bordeaux), Jacques Sizun (CHU Brest), Gilles Souet (ARS Centre), Gérard Thiriez (CHU Besançon), Pierre Tourneux (CHU Amiens), Marie Touzet (Hôpital de Port-Royal, Paris), Patrick Truffert (CHU Lille), Catherine Zaoui (CHG Valenciennes), Elodie Zana-Taieb (Hôpital de Port-Royal), and Claire Zores-Koenig (CHU Strasbourg).
